# Technical innovations in stroke rehabilitation – a survey for development of a non-invasive, brainwave-guided, functional muscle stimulation

**DOI:** 10.1186/s12883-022-02716-z

**Published:** 2022-05-25

**Authors:** Stefanie Liebl, Tim Tischendorf, Julia Winterlich, Tom Schaal

**Affiliations:** 1 Department of Public Health and Health Care Management, West Saxon University of Applied Sciences Zwickau, Kornmarkt 1, 08056 Zwickau, Germany; 2 University of Applied Science Mittweida, Albert-Schweitzer-Strasse 22, 09848 Mittweida, Saxony Germany

**Keywords:** Brain-Computer-Interface, EEG, Stroke, Motor rehabilitation, Functional electrical stimulation

## Abstract

**Background:**

Stroke is one of the most frequent causes of death in Germany and the developed countries. After a stroke, those affected often suffer particularly from functional motor restrictions of the upper extremities. Newer techniques such as the BCI-FES systems aim to establish a communication channel between the brain and external devices with a neuromuscular intervention. The electrical activity of the brain is measured, processed, translated into control signals and can then be used to control an application.

**Methods:**

As a mixed-methods design (exploratory design), eight guideline-based expert interviews were conducted first. For the quantitative expert survey, 95 chief physicians from the field of neuromedicine in rehabilitation facilities nationwide were subsequently invited to participate in an online survey.

**Results:**

In our data analysis, we found that doctors are largely open-minded towards new technical rehabilitation systems. In addition to the proper functioning of the system, they consider the understanding of the functionality and the meaningfulness of the system to be particularly important. In addition, the system should be motivating for individuals, generate meaningful movements, be easy to use, evidence-based and quick to set up. Concerns were expressed regarding the understanding of the system’s processes, especially in the acute phase after a stroke, as well as the excessive expectation of results from the system on the part of the persons. The experts named stroke patients in rehabilitation phase C, which is about mobilization and recovery, as well as all persons who can understand the language requirements as benefiting groups of people.

**Conclusion:**

The present study shows that more research should and must be done in the field of BCI-FES interfaces, and various development trends have been identified. The system has the potential to play a leading role in the rehabilitation of stroke patients in the future. Nevertheless, more work should be done on the improvement and implementation as well as the system’s susceptibility to interference in everyday patient life.

**Supplementary Information:**

The online version contains supplementary material available at 10.1186/s12883-022-02716-z.

## Context

Worldwide, stroke is the second most common cause of death and thus a major cost factor for health systems, as well as the main cause of disability in adulthood [1]. In 2020, 10.918 people (male: 4.326 female: 6.592) died of a stroke in Germany [2]. According to estimates, around 24.300–26.000 people in Germany experience a stroke every year [3]. It can be assumed that of all people currently suffering from a stroke, 200.000 experience a stroke for the first time and 65.000 suffer repeated strokes [4].

Up to 40% of those affected are exposed to permanent damage that leads to long-term restrictions in the activities of daily living (ADL) [1]. Particular problems occur in mobility, personal hygiene and independent dressing and eating [1]. Improving participation and ADLs is considered the primary goal of stroke rehabilitation, especially in the approximately six-month acute phase after a stroke event [5]. The survival rate 28 days after a stroke is about 78% [4].

Within the first month of the chronic phase, a quarter of people die after a first-time stroke [6]. Of those persons who survive the acute phase, 45% die within five years [6]. Of the remaining individuals, one-third are left with long-term disabilities and one-seventh are dependent on a nursing facility [6].

Spastic syndrome is developed in up to 40% of all stroke patients during the course, which is a common cause of persistent relevant disability as well as a major cause of ADL impairment [7]. Functional improvements of the upper extremities of stroke patients can be achieved through the use of Brain Computer Interfaces (BCI). It can be assumed that the temporal coupling of movement trials and visual and depth-sensitive feedback mediated by functional electrical stimulation (FES) promotes neuronal plasticity and favours motor rehabilitation [8]. However, lesion-induced plasticity after a cerebral insult must be distinguished from training-induced plasticity, as this effect also occurs in the healthy brain during learning processes [8].

**Fig. 1:**
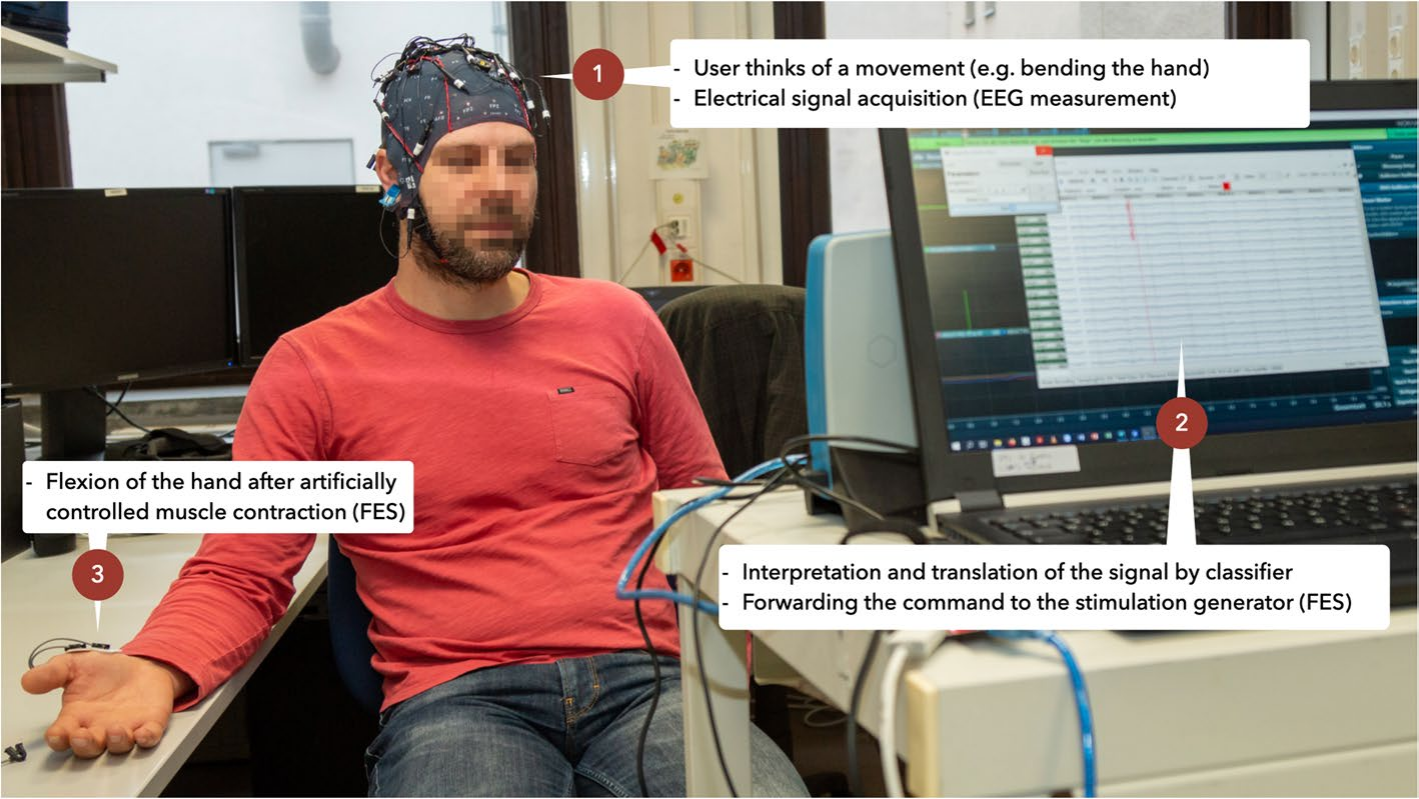
Experimental procedure of the BCI-FES system under laboratory conditions

Mostly, BCI systems are used to communicate the control of external devices [9]. The use in stroke rehabilitation is a new field of application [9]. In stroke rehabilitation, BCIs enable the control of FES to induce muscle contraction in the paralysed limb at the time of movement intention by detecting the appropriate brain signal [10]. FES is a technique that uses electrical currents to produce artificially controlled muscle contractions [11]. Although studies indicate a potential benefit of FES-based therapies of stroke rehabilitation, there is not yet clear evidence of a benefit of FES [11]. BCI is able to measure the desired activation of the motor cortex in real time, thus generating appropriate feedback [9]. BCI’s use EEG electrodes to establish a connection between the brain and a computer. When using BCI, individuals imagine a certain movement and the corresponding brain activity is recorded by EEG electrodes and sent to an amplifier. In the process, the classifier can be individually taught and thus better detect and interpret future movement intentions in the sense of machine learning (AI) [12]. If the correct movement is interpreted by the BCI’s classification algorithm, sensory feedback is provided using an electrical impulse via external devices (Fig. 1) [13]. BCI-FES systems aim to create a new communication channel between the brain and external devices without any neuromuscular intervention [14].

In this study, the remaining neuronal activity of the brain after a stroke is to be used by means of BCI to support the connections necessary for muscle contraction via neurofeedback. This procedure is feasible because stroke does not result in muscle damage, but functional limitations are caused by brain damage and communication interruptions as a result of the lesion of the upper motoneuron between the cognitive system and the muscles. Because of this, BCIs are becoming more and more of a focus in the rehabilitation of neuronal diseases such as stroke. The systematic review by Krueger et al. [10] showed that chronic stroke patients in particular benefit from the use of the BCI-FES systems. In five of 13 studies, training with BCI-FES systems improved motor function and mobility of the individuals’ extremities. In three of these studies, the difference to the control group was significant. Current studies give reason to believe that differences in effects exist especially with regard to cortical and subcortical lesions. The likelihood that motor activity can be measured by EEG is more likely in a subcortical stroke because there is still healthy tissue between the head surface and the lesion. The cortex remains intact despite the fact that the motor cortex no longer receives signals from the damaged subcortical brain areas [10].

The aim of the survey was to find out, in the field of accompanying medical research, the user readiness as well as the requirements and concerns about the system of BCI and FES. In addition, the aim was to identify the person group which, according to experts in the field of neuromedicine, would benefit most from this system.

## Methods

A structured interview guideline was first developed as a mixed-methods design (exploratory design). The subsequent expert survey served to explore the necessary requirements for a BCI-FES system from the user’s perspective and was conducted in 2020. This was associated with a target group-oriented product development that can establish itself on the market [15]. Following common qualitative sampling strategies, care was taken to ensure that the group was as heterogeneous as possible when selecting the interview partners [16]. The target groups were experts from the professional groups of medicine (*n* = 5 doctors), physiotherapy (*n* = 2 physiotherapists) and nursing (*n* = 1 nurse) of a hospital with a supraregional stroke unit. After pre-selection, the interviewees who had agreed in principle to participate received information material on the topic of the interview. All respondents gave their consent to participate in the study after prior information. The data was analysed according to Mayring’s systematic, rule-guided procedure. The focus was on the development of a category system. Through inductive category definition, the categories were derived directly from the material without referring to previously defined theoretical concepts [17]. The categories were created independently by two researchers and then compared with each other. The deductively formed categories and inductively formed subcategories were coded during the analysis of the interview material [18].

**Fig. 2:**
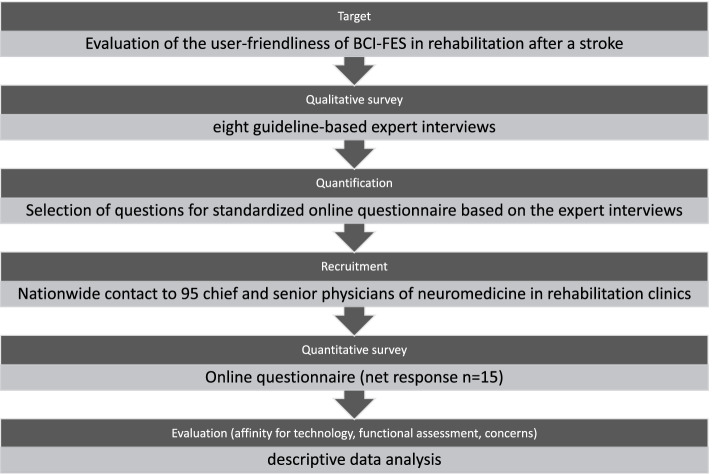
Mixed-methods design (exploratory design) based on Krueger et al. 2020 Rehabilitation after stroke

Subsequently, the needs identified were examined in a quantitative survey with regard to their generalisability (Fig. 2). Results of the qualitative sub-study were included in the hypothesis development and questionnaire construction of the quantitative sub-study [19]. Based on the qualitative survey, the questionnaire of the quantitative survey was developed and expanded to include the area of technology affinity [20].

The questionnaire comprised 20 items and consisted mainly of closed questions. Sociodemographic data, general questions on technology affinity, general questions on rehabilitation practice, BCI and FES, modes of functioning of the system in practical use, concerns about use of BCI and FES, and benefiting person groups were collected (e-supplement). The online survey was functionally and linguistically checked and adapted in a pretest.

All rehabilitation hospitals with a neuromedicine department nationwide were included and 95 chief physicians or senior physicians were invited to participate in the online survey via their service e-mail address. There were no invalid e-mail addresses. The contact data were obtained via a manual search at www.rehakliniken.de. In addition to the personalised participation link to the survey, information material on the survey topic was sent out. In three follow-up actions, reminders were addressed to people who had not yet accessed the online questionnaire by that time.

The survey took place online from June to August 2021 and the data was collected via www.soscisurvey.de free of advertising exclusively for scientific purposes.

In addition to the objectives, the homepage of the survey referred to voluntary participation and anonymous data processing, and participants were asked to give their consent to data protection before further processing. A comprehensive data protection concept was also available for download. Applicable guidelines to ensure good scientific practice were observed [21].

## Results

The link to the survey was clicked on by 32 doctors regardless of whether the questionnaire was subsequently closed again, only the introduction was read or the questionnaire was processed further. The net sample size consisted of 15 participants. A further 17 doctors had abandoned the questionnaire prematurely.

Descriptive data analysis was performed using SPSS 26 (Statistical Package for the Social Sciences). All respondents were classified as neurologists. Only male doctors participated and were classified in the age groups 40–49 years (*n* = 3), 50–59 years (*n* = 9) and 60 years and older (*n* = 3). Two of the 15 respondents reported professional experience of between 16 and 20 years and 13 of more than 20 years. Measured in terms of all individuals treated by the doctors, they put the proportion of stroke patients to be treated at $\overline{x }$=53.3% (SD ± 17.2).

**Fig. 3:**
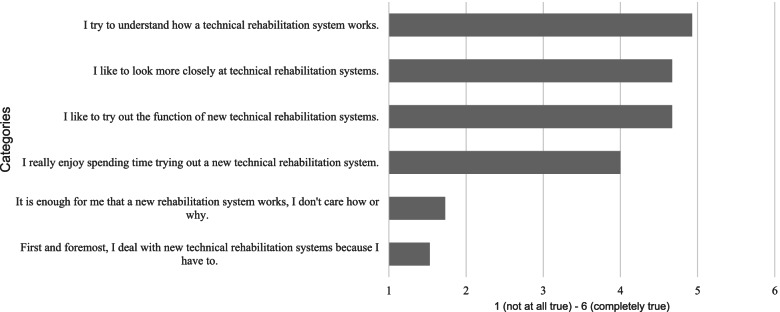
Questions on affinity with technology. The doctors were asked to indicate their level of agreement with statements about technology affinity

Based on the ATI scale [20], it was found that doctors are largely open to new technical rehabilitation systems. The respondents not only consider it important that the system works. They also want to understand how the system works and why it makes sense and are willing to invest their time in trying out the new functions of technical rehabilitation systems (Fig. 3).

At the time of the survey, the majority of doctors stated that they use physiotherapy (15/15), occupational therapy (15/15), speech therapy (15/15) as well as neuropsychology (14/15) and sports therapy (13/15) in their current everyday rehabilitation of stroke patients. Work therapy, on the other hand, was only mentioned by six doctors.

The question about innovative technologies that are already used in everyday rehabilitation gave a differentiated picture. While two of the 15 doctors do not use any innovative technologies and another three participants abstained, the remaining ten respondents stated that they use a whole range of innovative rehabilitation systems in their everyday practice. These include gait (15/15), balance (14/15), arm (14/15), hand (14/15), leg (12/15) and knee (10/15) rehabilitation systems, as well as computer-assisted systems in speech and language therapy (open responses). Furthermore, the use of exoskeleton and virtual reality systems was increasingly indicated, which were used in combination with video and game-guided, device-based therapy for arm and hand rehabilitation. In addition to FES, transcranial direct current stimulation and transcranial magnetic stimulation systems were listed, which, like the BCI-FES technology described, represent non-invasive, neurophysiological methods in rehabilitation.

Six out of 15 physicians were aware of the BCI system in combination with FES, nine denied this. Seven doctors stated that they had had their first contact with the system during their previous work. Another seven respondents said they had no contact with the system and one doctor also answered in the negative, although he had already had some experience with similar systems. All respondents could imagine working with the technical rehabilitation system BCI in combination with FES. Eight out of 15 participants could imagine working with the system without further reservations and seven other doctors with reservations.

**Fig. 4:**
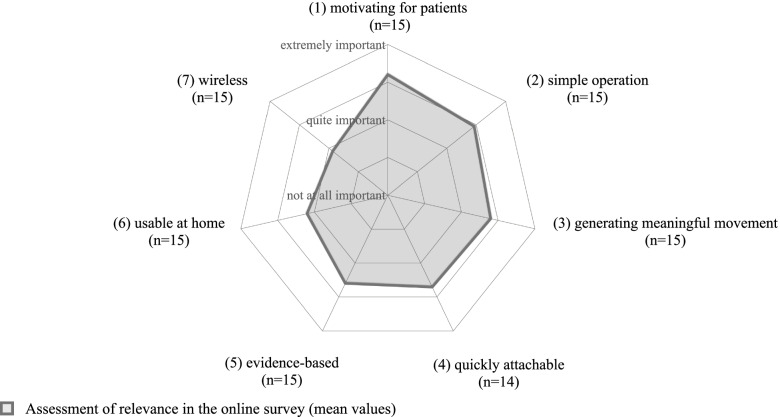
Explored functions. The doctors were asked to indicate which of the seven application-related requirements for the BCI-FES system were of particular importance to them

Seven application-related requirements for the BCI system in combination with FES were explored in the qualitative expert survey and quantified in the online survey with regard to their relevance (Fig. 4).

Additional important functions mentioned by the participants were an affordable duration of the treatment unit, measurability of the promotion of neuroplasticity and a technique that can be easily adapted to the specific problems of the person. According to the experts, these functions should also make it possible to make low-threshold effects comprehensible and to enable transferability of the training beyond sector boundaries. In other words, to make it possible to teach the system oneself in order to adapt it more efficiently to the individual’s needs and to maximize the success of the therapy. Other important features mentioned were the durability and robustness of the system and an affordable price.

**Fig. 5:**
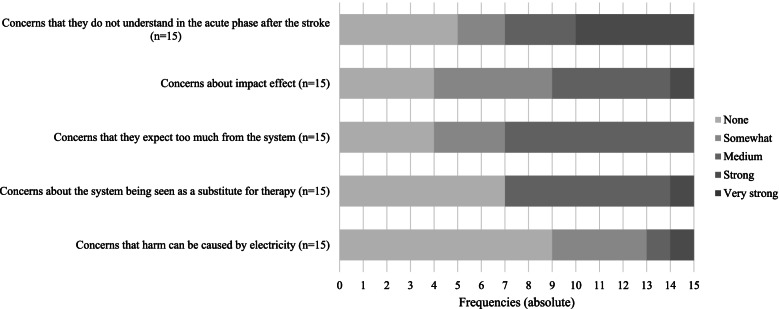
Concerns. The doctors were asked to indicate the degree of their concerns regarding the five aspects listed

Clinicians were also asked to rate their concerns about selected aspects of the BCI system in combination with FES (Fig. 5).

**Fig. 6:**
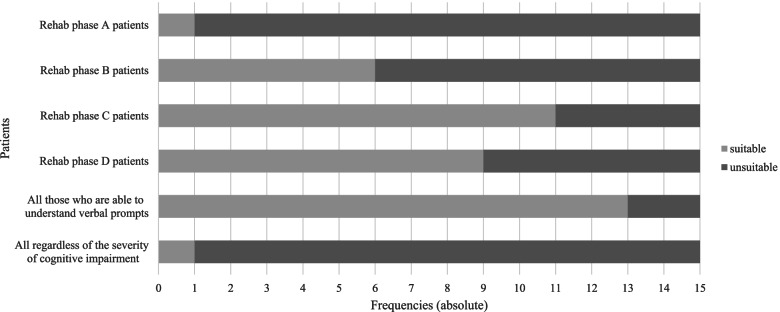
Person groups. The doctors were asked to indicate their level of agreement with the use of the BCI-FES system in the listed person groups

Stroke patients are divided into different phases using the Early Rehabilitation Barthel Index [22]. Phase A includes acute care, in which the individual is cared for on a stroke unit, an intensive care unit or a normal ward. In phase B, early rehabilitation begins, in which intensive treatment and rehabilitation with medical and therapeutic focal points are carried out. In phase C, the continuing rehabilitation, the person no longer needs so much help in coping with everyday life, so the focus here is on mobilization and restoring independence. Purely medical rehabilitation ends with phase D, the follow-up treatment, in which the focus is on reducing existing disabilities [23]. As a result of the survey, a differentiated assessment of the use of the system within these rehabilitation phases could be determined. For all individuals, regardless of the severity of the cognitive impairment, the system is suitable for one of the 15 doctors. One doctor agreed to use the system for phase A persons, six doctors for phase B persons, eleven doctors for phase C persons and nine doctors for phase D persons. In addition, 13 out of 15 of the respondents considered the system of BCI in combination with FES suitable for all individuals who are able to understand speech prompts (Fig. 6).

Individuals suffering from Complex Regional Pain Syndrome (CRPS) have also been reported as possible beneficiaries. CRPS is a life-changing condition that usually affects the extremities following trauma or nerve injury [24]. In addition, persons have been reported as suffering from other acute acquired brain injuries or diseases of the central nervous system, such as traumatic brain injury (TBI) or multiple sclerosis (MS). TBI refers to any injury to the skull that is associated with damage to the brain [25]. MS is an autoimmune disease of the central nervous system characterized by chronic inflammation, demyelination, gliosis and neuronal loss [26].

## Discussion

Our results suggest that post-stroke rehabilitation through BCI-FES is a sustainable approach that opens new possibilities for stroke rehabilitation [10]. The 15 doctors interviewed are open-minded and interested in new innovative rehabilitation technologies, whereby these should be both functional and understandable in their meaningfulness and function. However, this thoroughly positive attitude of the doctors is only reflected to a limited extent in the varying degree of use of the systems in everyday rehabilitation. It becomes clear that a large number of different rehabilitation systems are already used in everyday practice [14]. Among them, a construct very similar to the BCI-FES system was mentioned, based on transcranial direct current and magnetic current stimulation systems, which also represents a non-invasive, neurophysiological method in rehabilitation. Almost half of the doctors interviewed had already had first contact with BCI-FES systems and all participants could imagine working with such a system. The doctors are concerned about the plasticity potential of the technique, patient specificity and the easiness of comprehension of the intervention. Due to insufficient evidence in the literature, further research needs to be done on this issue.

However, our results from the expert interviews already indicate that the system in its current form is not sufficiently practicable for use in the daily rehabilitation routine of a stroke unit. Excessively long preparation times interfere with the smooth running of the therapy unit. The complex electrode arrangement makes it difficult to carry out the therapy through time-limited units, which is also confirmed by the literature research [10, 11].

The previous evidence base in this area is very thin, so it is not yet possible to recommend BCI systems as standard therapy. Currently, no superiority of the system can be established. However, there are different options for using the BCI-FES system. In this work we have limited ourselves to this form of detection. Due to the weak evidence already mentioned, the system cannot currently be regarded as a standard therapy. However, it can be considered as a new therapeutic approach for which the present study aims to contribute to its applicability in practice.

Nevertheless, the use of BCI-FES systems on individuals raises some issues about which medical professionals are divided. Concerns about harm to the person from the applied current were judged to be rather unproblematic. The risk that the method could be viewed as a substitute for therapy also appeared unproblematic. In order to counteract this possible contraindication, particular attention could be paid to the slow increase in current pulses. Furthermore, care could be taken to ensure that skin-friendly adhesive pads are used in order to minimize the risk of skin irritation Moderate to strong concerns were expressed about the effect and the person’s expectations of the system being too high. Clinicians were divided about the effect and the person’s understanding of the system in the acute phase immediately after stroke [14]. Responses ranged from no concerns to moderate to strong concerns.

These concerns should be addressed in future work through the use of the system [14]. One particularly important issue is educating the individual about the system. According to a doctor in the study, concerns about the technology, applicability, opportunities, and risks can be minimized through intensive educational efforts with trained personnel. This can maximize the acceptance and motivation of individuals. With regard to increasing motivation, the measurability of therapy success should be further developed in the future. The possibility of training at home in the form of digital training could also increase motivation and user-friendliness. On a positive note, the survey made it possible to narrow down the scope of intervention.

In addition, experts believe that the system is not suitable for every stroke rehabilitation phase. The system is considered useful only in rehabilitation phases C and D and for people who can understand the language requirements of the system [22]. People suffering from CRPS were also mentioned by clinicians as other beneficiary groups [24].

A possible limitation of the study results may be response bias [19]. According to this, the values reported by the respondents could systematically deviate from the actual values, in that only doctors who are particularly interested in this topic or have an interest in the science responded.

## Conclusion

The present study shows that more research should and must be done in the field of BCI-FES interfaces. The system has the chance to play a leading role in the rehabilitation of stroke individuals in the future. Nevertheless, more work should be done on improving and implementing the system in everyday person care. Only in this way can a user-friendly and user-defined therapy be guaranteed by BCI-FES systems, as negative aspects were also broken down in addition to many positive aspects.

## Supplementary Information

**Additional file 1.**

**Additional file 2.**

## Data Availability

All data generated or analysed during this study are included in the supplementary information files.

## Abbreviations

ADL: Activities of daily living
AI: Artificial intelligence
ATI-Scale: Affinity for technology interaction scale
BCI: Brain Computer Interfaces
CRPS: Complex Regional Pain Syndrom
E-Supplement: Electronic-Supplement
EEG: Electroencephalogram
Et al: Et alii
FES: Functional electrical stimulation
MS: Multiple sclerosis
n: Number of cases
SD: Standard deviation
TBI: Traumatic brain injury
x̅: Mean value
