# Supplementary material for: Technical innovations in stroke rehabilitation – a survey for development of a non-invasive, brainwave-guided, functional muscle stimulation
Source: BMC Neurol. 2022 May 25;22:194. doi: 10.1186/s12883-022-02716-z (PMC9131555; doi:10.1186/s12883-022-02716-z)
Supplement: Supplementary file 2 — Additional file 2. [file 12883_2022_2716_MOESM2_ESM.pdf]

Brain-Computer Interface (BCI) in conjunction with Functional Electrical Stimulation (FES)

Dear doctors,

Thank you for your interest in our study.

Strokes are considered the second leading cause of death worldwide and are among the most common causes of severe disability in adulthood. Effective rehabilitation can have a long-term impact on the quality of life of those affected.

To alleviate neurodegenerative diseases, more research is therefore being done on synthetic replacement of brain-muscle connections by brain-computer interfaces (BCIs). BCIs aim to create a communication channel between the brain and external devices without any neuromuscular intervention.

In this novel rehabilitation method, patients imagine a certain movement, whereby the corresponding brain activity is recorded via EEG electrodes (electroencephalography) in conjunction with the BCI, analysed and used in real time.
The movement presented by the patient is interpreted by a classification algorithm of the BCI and converted into sensory feedback by means of functional electrical stimulation (FES) via external devices.
Functional electrical stimulation (FES) is a technique that uses electrical currents to create artificially controlled muscle contractions.

The research project "Technical innovations in stroke rehabilitation - development of a non-invasive, brainwave-controlled, functional muscle stimulation", funded by the Saxon State Ministry of Science, Culture and Tourism (SMWK), is concerned with the further development of an innovative rehabilitation technology. In this context, we are researching the user readiness as well as the application possibilities of brain-computer interfaces in connection with functional electrical stimulation (FES).

For the results to be representative, it is important that as many people as possible take part in the ten to twelve minute survey. The questions in the questionnaire relate to your opinion. We would therefore like to ask you to fill in the questionnaire personally. Your information will of course be treated anonymously and confidentially. Participation in the study is voluntary, and you will not suffer any disadvantages if you refuse or cancel the survey.

If you have any questions, please do not hesitate to contact us for further information. Please contact

Survey questions:
- Ms. S. Liebl, E-Mail: stefanie.liebl@fh-zwickau.de
- Mr. T. Tischendorf, E-Mail: tim.tischendorf.khx@fh-zwickau.de

Data protection: Mr. M. Süß, E-Mail: marcel.suess@fh-zwickau.de

---

Page 02

Data protection: Your information cannot be linked to your person. The anonymised data from the survey will be stored for scientific purposes for at least ten years after the end of the project. Third parties will not have access. You can reach the WHZ data protection officer, Mr Marcel Süß, at: (0375) 536- 1110 or marcel.suess@fh-zwickau.de. You can find the detailed data protection declaration here.

Declaration of consent: I declare that I have taken note of the information on data protection and agree to the anonymous storage and processing of my data as well as the publication of the results in summarised form. Due to the anonymous storage, a subsequent revocation of my information is not possible.

- ◯ I explicitly agree to the data processing
- ◯ I do not agree and would like to leave the survey

1. What is your profession?

- Doctor for neurology
- Doctor of another speciality, namely

2. How many years of professional experience do you have?

- Under 5 years
- 5-10 years
- 11-15 years
- 16-20 years
- Over 20 years
- Not specified

3. How old are you?

- Under 18 years
- 18-29 years
- 30-39 years
- 40-49 years
- 50-59 years
- Over 60 years
- Not specified

4. What gender are you?

- Male
- Female
- Divers
- Not specified

5. Please indicate your level of agreement with the following statements:

Note: Technical rehabilitation systems refers to computer and app-based rehabilitation techniques.

	Not true at all	Largely not true	Rather not true	Rather true	Largely true	Totally true
I like to look more closely at technical rehabilitation systems.	○	○	○	○	○	○
I like to try out the function of new technical rehabilitation systems.	○	○	○	○	○	○
First and foremost, I deal with new technical rehabilitation systems because I have to.	○	○	○	○	○	○
I really enjoy spending time trying out a new technical rehabilitation system.	○	○	○	○	○	○
It is enough for me that a new rehabilitation system works, I don't care how or why.	○	○	○	○	○	○
I try to understand how a technical rehabilitation system works.	○	○	○	○	○	○

6. Which disease patterns do you care for in total?

Multiple selection possible

- ☐ Strokes
- ☐ Brain haemorrhages
- ☐ Craniocerebral trauma
- ☐ Multiple sclerosis
- ☐ Guillain-Barré syndrome
- ☐ Muscle diseases
- ☐ Infections of the nervous system
- ☐ Tumours of the nervous system
- ☐ None of the above
- ☐ Others: ______

7. In terms of all the patients you treat: What is the percentage of stroke patients?

Please estimate.

______

8. Which rehabilitation techniques do you currently use in the everyday rehabilitation of stroke patients?

Multiple selection possible

- ☐ Physiotherapy
- ☐ Occupational therapy
- ☐ Speech therapy
- ☐ Neuropsychology
- ☐ Sports therapy
- ☐ Occupational therapy
- ☐ Others:

9. Which innovative technical rehabilitation systems do you know?

Computer or robot-based systems; multiple selection possible

- ☐ Gait rehabilitation systems
- ☐ Balance rehabilitation systems
- ☐ Arm rehabilitation systems
- ☐ Hand rehabilitation systems
- ☐ Leg rehabilitation systems
- ☐ Knee Rehabilitation Systems
- ☐ More:

10. Which innovative technologies do you use in your everyday rehabilitation work?

The following figure describes how brain-computer interface (BCI) works in conjunction with functional electrical stimulation (FES).

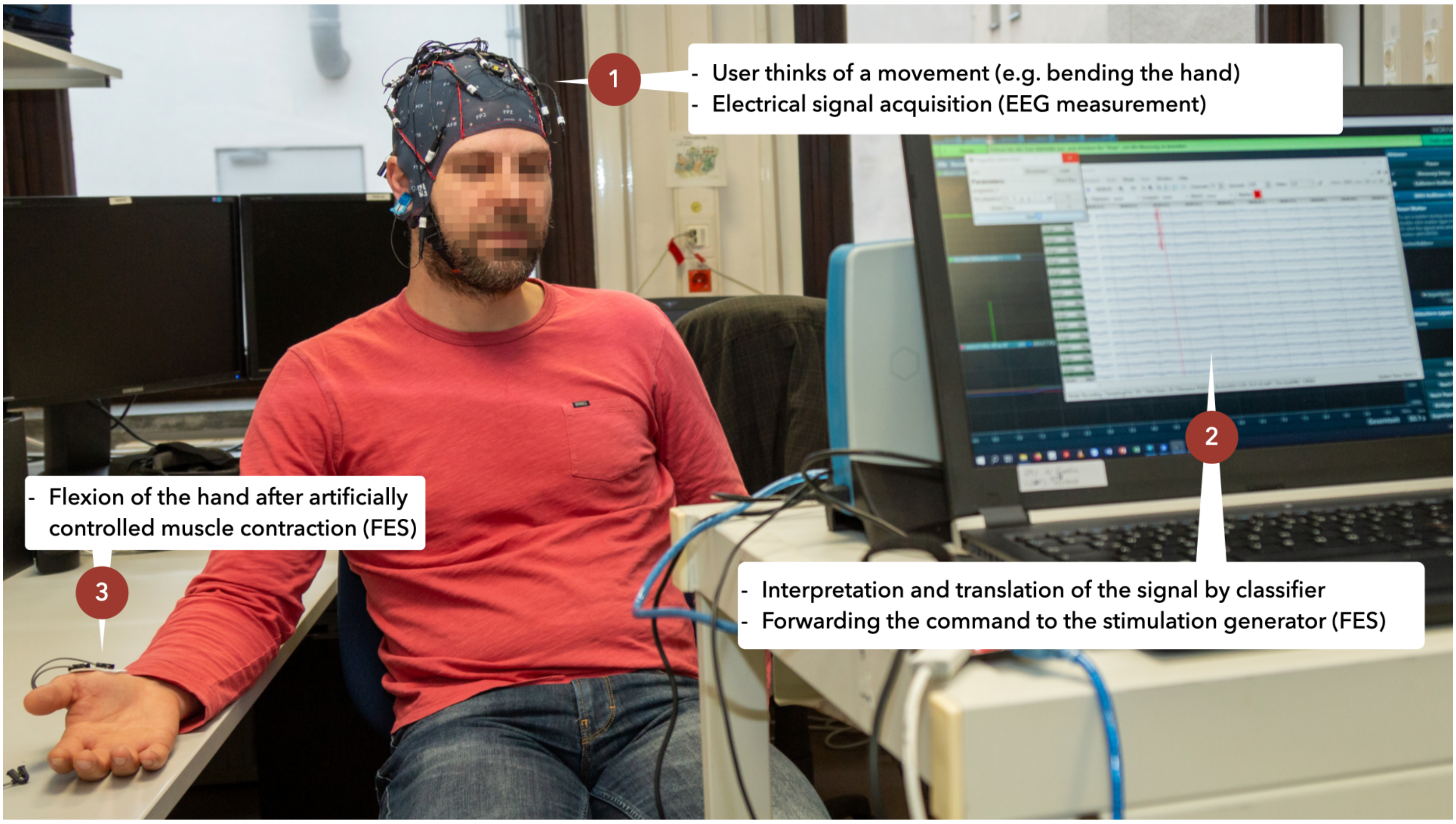

11. Have you had any contact with brain-computer interface during your work?

- ◯ Yes
- ◯ Nop
- ◯ No, but with similar systems

12. Do you know brain-computer interface in connection with functional electrical stimulation?

- ◯ Yes
- ◯ No

13. Could you imagine working with the Brain-Computer-Interface rehabilitation system in conjunction with functional electrical stimulation?

- ◯ Yes, in any case
- ◯ Yes, with reservations
- ◯ No

14. How important would the following functions of the above system be to you?

Brain-computer interface in conjunction with functional electrical stimulation

	Not important at all	Somet hing importa nt	Quite importa nt	Very importa nt	Extremely important
It must be evidence-based (proven effectiveness).	◯	◯	◯	◯	◯
It must be easy to use.	◯	◯	◯	◯	◯
It must be quick to put on.	◯	◯	◯	◯	◯
It has to be wireless.	◯	◯	◯	◯	◯
It would have to produce meaningful movement.	◯	◯	◯	◯	◯
It must be motivating for the patients.	◯	◯	◯	◯	◯
It must be usable at home by the relatives.	◯	◯	◯	◯	◯

15. What other functions are important to you?

If you can't think of any, please move on to the next question.

16. How strong are your concerns about patients using brain-computer interface in conjunction with functional electrical stimulation?

	None	Somewhat	Medium	Strong	Very Strong
Concerns about impact effect	○	○	○	○	○
Concerns that they do not understand in the acute phase after the stroke	○	○	○	○	○
Concerns that they are expecting too much from the system	○	○	○	○	○
Concerns about the system being seen as a substitute for therapy	○	○	○	○	○
Concern that damage can be caused by electricity	○	○	○	○	○

17. Are there any further concerns for you?

Please name them.

18. For which stroke patients do you think it would be useful to use brain-computer interface in combination with functional electrical stimulation?

Multiple selection possible

- ☐ For all patients regardless of the severity of cognitive impairment
- ☐ For patients in rehabilitation phase A
- ☐ For patients in rehabilitation phase B
- ☐ For patients in rehabilitation phase C
- ☐ For patients in rehabilitation phase D
- ☐ For all patients who are able to understand verbal prompts
- ☐ For none of the designated

19. Suppose you have to decide on a patient group. Which one do you think would benefit most from brain-computer interface combined with functional electrical stimulation?

This question is very important to understand which patients are most likely to benefit from the system from a practice perspective.

Only one answer option

- ○ All patients regardless of the severity of cognitive impairment
- ○ Rehab phase A patients
- ○ Rehab phase B patients
- ○ Rehab phase C patients
- ○ Rehab phase D patients
- ○ All patients who are able to understand verbal prompts
- ○ None of the designated

20. For which patients besides stroke patients would rehabilitation also be suitable in your opinion?

Page 11

Thank you for your interest.

You can contact the following persons if you have any questions:

- Questions about the research project: Prof. Dr. Schaal, E-Mail: tom.schaal@fh-zwickau.de
- Survey questions: Stefanie Liebl, E-Mail: stefanie.liebl@fh-zwickau.de
- Questions about data protection: Marcel Suess, E-Mail: marcel.suess@fh-zwickau.de

Finanzierung: Diese Maßnahme wird mitfinanziert mit
Steuermitteln auf Grundlage des vom sächsischen Landtag
beschlossenen Haushaltes.

SACHSEN

Last Page

Thank you for your participation!

We would like to thank you very much for your help.

Your answers have been saved, you can now close the browser window.

Tim Tischendorf, West Saxon University of Applied Sciences Zwickau – 2021
